# Timing of transcutaneous acupoint electrical stimulation for postoperative recovery in geriatric patients with gastrointestinal tumors: study protocol for a randomized controlled trial

**DOI:** 10.3389/fmed.2025.1497647

**Published:** 2025-03-05

**Authors:** Weijuan Yin, Fang Fang, Yan Zhang, Lijuan Xi

**Affiliations:** ^1^Department of Gastrointestinal Surgery, The Affiliated Hospital of Yangzhou University, Yangzhou University, Yangzhou, China; ^2^The Nursing Department, Northern Jiangsu People’s Hospital, Yangzhou, China; ^3^Jiangsu Taizhou People's Hospital, Taizhou, China; ^4^School of Nursing and School of Public Health, Yangzhou University, Yangzhou, China

**Keywords:** transcutaneous acupoint electrical stimulation, postoperative cognitive dysfunction, geriatric patients, gastrointestinal tumor, randomized controlled trial

## Abstract

**Purpose:**

To develop a study protocol for determining the optimal timing of Transcutaneous Electrical Acupoint Stimulation (TEAS) to enhance postoperative recovery in elderly patients. The study aims to evaluate different timing strategies for TEAS administration and their effects on postoperative outcomes, with the goal of improving clinical practices and guiding future research.

**Methods:**

A total of 266 geriatric patients who underwent radical resection of gastrointestinal tumors will be divided into seven groups: one control group (receiving standardized perioperative management), one sham intervention group (receiving TEAS treatment without electrical stimulation), and five intervention groups (receiving TEAS at different time intervals). The intervention groups will receive TEAS at bilateral Neiguan (PC6) and Zusanli (ST36) acupoints. The TEAS treatment will employ an altered frequency of 2/100 Hz with disperse-dense waveforms and an adjustable intensity, ensuring the stimulation remains below 10 mA and within a tolerable range for the patient. The device will output an asymmetrical biphasic pulse wave, with a pulse width of 0.2 ms ± 30%, based on electromagnetic compatibility basic performance testing. The primary outcome will assess changes in cognition, measured using neuropsychological tests administered preoperatively and 3 days postoperatively, as well as the Telephone Interview for Cognitive Status-Modified (TICS-m) at 1, 3, and 6 months postoperatively. Secondary outcomes will include preoperative and 3-day postoperative measurements of interleukin-6 (IL-6), S100 calcium-binding protein β (S100β), tumor necrosis factor alpha (TNF-*α*), insulin-like growth factor 1 (IGF-1), and C-reactive protein (CRP). Additional data will be collected on the time to postoperative exhaust, defecation, eating, and the first postoperative ambulation. Numeric Rating Scale (NRS) scores will be recorded before and on the third day after the operation, alongside Activities of Daily Living (ADL) and Braden scale scores, which will be assessed before the operation and at the time of discharge.

**Discussion:**

This protocol aims to determine the optimal timing of TEAS for improving postoperative recovery in geriatric patients with gastrointestinal tumor.

**Clinical trial registration:**

ClinicalTrials.gov, identifier NCT05482477.

## Introduction

Transcutaneous electrical acupoint stimulation (TEAS) is a novel therapy that involves applying low-frequency pulse currents to specific acupoints on the skin’s surface. This therapy combines the benefits of both acupuncture and transcutaneous electrical nerve stimulation (TENS) to achieve therapeutic outcomes (1). TEAS is non-invasive, easy to administer, and well-accepted by patients (2).

Postoperative cognitive dysfunction (POCD) is a common central nervous system complication in cancer patients, with an incidence ranging from 8.9 to 46.1% (3). It primarily manifests as memory impairment, decreased information processing capacity, and declines or disruptions in attention, perception, abstract thinking, executive function, language, and motor skills (4, 5). POCD can be challenging to diagnose but may persist for months or even become a permanent condition (6), significantly impacting patients’ postoperative recovery, reducing their quality of life, increasing mortality rates, and burdening both family and medical social resources, thereby contributing to the overall economic and social burden (7, 8).

Studies have demonstrated that TEAS treatment administered 10–30 min before entering the operating room or continuously throughout the surgical procedure can reduce the incidence of POCD in elderly patients (9–12). TEAS administered either preoperatively or in combination with postoperative treatment has been shown to significantly improve postoperative cognition (13). Our research has demonstrated that perioperative TEAS can effectively reduce the postoperative inflammatory response and lower the incidence of POCD in geriatric patients (14).

Furthermore, studies have indicated that TEAS can alleviate postoperative pain in patients (15), reduce the time to the first flatus and defecation (16), and expedite post-surgical ambulation (17). In our previous study, we observed a significant difference in the Braden scale scores between the pre- and postoperative TEAS groups and the control group at the time of discharge, while the Activities of Daily Living (ADL) scores remained similar (18).

TEAS treatments administered at various times have demonstrated improvements in postoperative cognition. However, it is important to note that long-term electroacupuncture treatment may lead to the development of a ‘tolerance effect,’ which triggers a negative feedback mechanism and reduces the number of receptors, potentially diminishing its therapeutic efficacy (19). Therefore, this study aims to outline the protocol for a research project currently in the planning stage, designed to determine the optimal timing of TEAS for enhancing postoperative recovery in elderly patients.

## Methods and analysis

### Design

**Figure 1:**
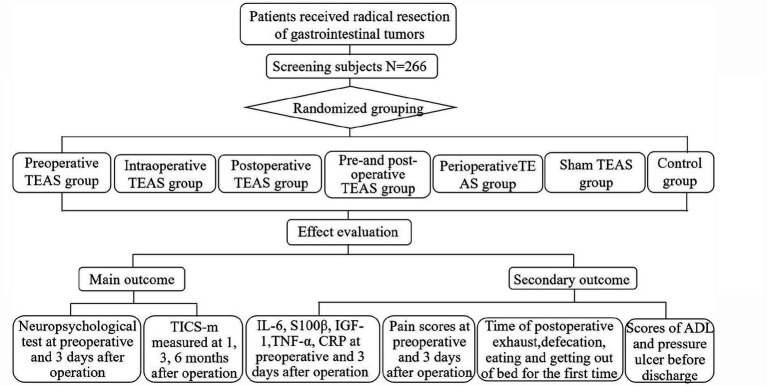
Flow chart of the study protocol. TEAS, transcutaneous electrical acupoint stimulation; TICS-m, Telephone Interview for Cognitive Status-Modified; IL-6, Interleukin-6; S100β, S100 calcium protein β; IGF-1, Insulin-like growth factor-1; TNF-α, tumor necrosis factor, CRP, C-reactive protein; NRS, numeric rating scale; ADL, Activities of Daily Living.

From January to December 2025, we will conduct a prospective, single-center, single-blind, randomized controlled trial at Northern Jiangsu People’s Hospital. Figure 1 illustrates the study flowchart.

### Patient population and setting

The equation for sample size calculation, *n* = (Z*_α/2_* + Z*_β_*)^2^ × 2*σ*^2^/δ^2^ where σ represents the average score of the Mini-Mental State Examination (MMSE) evaluated on the third day after the operation between the two groups. Based on previous pre-experimental data, this study employs a two-sided test with *α* = 0.05 and a test efficiency of (1-β) = 0.9, resulting in Z_α/2_ = 1.96, Z_β_ = 1.282. *σ* denotes the standard deviation, while *δ* represents the mean difference of MMSE scores on the third day after the operation between the intervention and control groups. According to the pre-experiment results, σ is 2.35, δ is 2.0, and considering a 30% increase in the sample size, each group requires 38 patients. Therefore, a total of 266 patients will be sequentially enrolled after meeting the eligibility criteria, with the attending doctor informing each patient and obtaining their informed consent. This study adheres to the principles of the Declaration of Helsinki.

To expand our pool of research subjects, we will promote recruitment on the hospital’s official account. Throughout the intervention, patients will retain the final decision-making authority regarding their participation in the trial.

### Eligibility criteria

Inclusion criteria:

1. Patients aged 60 years or older.
2. Patients diagnosed with gastrointestinal tumors who undergo radical resection under general anesthesia at Northern Jiangsu People’s Hospital.
3. American Society of Anesthesiology (ASA) classification I ~ III.
4. Preoperative Tilburg frailty scale score less than 5 points.
5. Preoperative D-dimer within normal limits.
6. Participants have completed a formal education program lasting more than 5 years (20).
7. Patients who are willing and able to give informed consent and comply with the study protocol.

Exclusion criteria:

1. Preoperative cognitive dysfunction or a history of cognitive dysfunction, dementia, or delirium.
2. History of severe depression, schizophrenia, or other mental or neurological disorders, or those currently taking antipsychotic or antidepressant medications.
3. Patients with severe hearing or visual impairment without the use of assistive devices.
4. Patients with communication difficulties.
5. Male patients with an average daily pure alcohol intake ≥61 g or female patients with an average daily intake ≥41 g (21).
6. Patients who have undergone surgical treatment within the past 3 months or who have been hospitalized preoperatively for more than 3 months.
7. Patients with severe heart, liver, or renal failure.
8. Patients with hypoxemia (blood oxygen saturation < 94%) for more than 10 min during surgery.
9. Patients admitted to the ICU postoperatively.
10. Patients who withdraw or die due to noncooperation or unforeseen circumstances.
11. Patients participating in other clinical studies that may interfere with the results of this study.
12. Patients who undergo emergency surgery.
13. Patients with a history of acupuncture treatment.

### Randomization and blinding

This is a single-blind study. To ensure random allocation, serial numbers from 1 to 266 will be placed in sealed opaque envelopes for the patients. Specifically, numbers 1–38 will correspond to the preoperative TEAS group, numbers 39–76 will be assigned to the intraoperative TEAS group, numbers 77–114 will belong to the postoperative TEAS group, numbers 115–152 will be designated for the pre- and postoperative TEAS group, numbers 153–190 will be allocated to the perioperative TEAS group, numbers 191–228 will be assigned to the sham TEAS group, and numbers 229–266 will be assigned to the control group. The randomization process will be conducted by a study administrator who is not directly involved in the study.

### Patient and public involvement

Patients or members of the public were not involved in the design, conduct, reporting, or dissemination of this research. However, study participants will have access to the study results through our social media channels.

### Intervention

The control group will receive standard perioperative care, which includes preoperative health education, an optimized anesthesia plan, intraoperative heat preservation measures, and minimized surgical trauma.

The intervention groups and the sham intervention group will undergo transcutaneous electrical acupoint stimulation (TEAS) in addition to the standard perioperative care provided to the control group. Specifically, electrodes will be attached to the skin surface of the acupoint and connected to the transcutaneous electrical stimulators (SDZ-III, Suzhou Medical Technology, Suzhou, China). The device will operate at a frequency of 2/100 Hz, utilizing disperse-dense waveforms. The stimulation intensity will be adjusted to a level that the patient can tolerate, but will not exceed 10 mA. The output will consist of an asymmetrical biphasic pulse wave, with a pulse width of 0.2 ms ± 30% (based on electromagnetic compatibility basic performance testing).

Considering the specificities of gastrointestinal organs and the principles of “Xingnao Kaiqiao Acupuncture” in traditional Chinese medicine (22), two acupuncture points have been selected: bilateral Neiguan (PC6) and Zusanli (ST36). Traditional Chinese medicine posits that gastrointestinal surgeries, like those causing physical trauma, may lead to essence deficiency, loss of “Qi, “stagnation, and blood stasis. Therefore, postoperative recovery should focus on eliminating blood stasis, promoting collateral circulation, and restoring visceral “Qi” (23).

**Figure 2:**
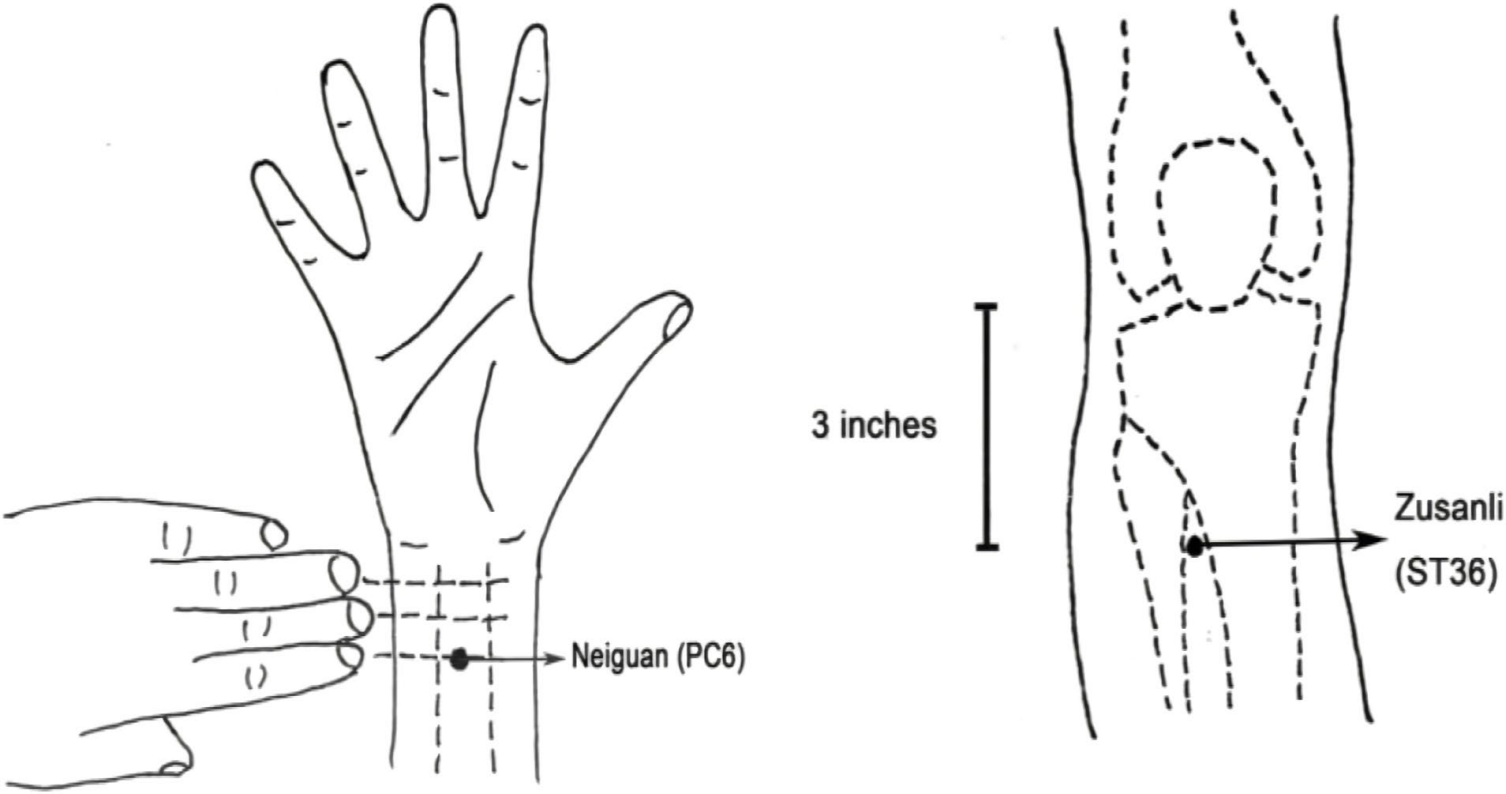
Location of acupoints.

PC6 is located on the inner side of the forearm, approximately 3 cm proximal to the transverse carpal crease, positioned between the palmaris longus tendon and the radial flexor carpi tendon (24). PC6 is an acupoint on the Pericardium Meridian of Hand Jueyin and is closely related to mental activities, cognitive function, and brain function (25). ST36 is located 3 inches below the outer side of the knee (26). ST36 is considered one of the primary acupoints on the Stomach Meridian of Foot Yangming and is associated with functions such as regulating the spleen and stomach, tonifying the middle, replenishing Qi, and promoting meridian flow (27). Please refer to Figure 2 for the precise location of these acupoints. During TEAS treatment, the acupoints on the same side will be grouped together for stimulation.

The timing of TEAS treatments for each group were as follows:

1. Preoperative TEAS group: 30 min before anesthesia (28).
2. Intraoperative TEAS group: From 30 min before anesthesia to the end of surgery (29).
3. Postoperative TEAS group: For 7 consecutive days after the operation, once daily in the morning, with each session lasting 30 min (30).
4. Pre-and postoperative TEAS group: From 1 day before the operation until 7 days after, once daily in the morning, with each session lasting 30 min (30).
5. Perioperative TEAS group: 30 min before anesthesia until the end of surgery, 1 day before the operation, and on the 1st, 2nd, and 3rd days after surgery in the morning, each session lasting 30 min (14).
6. Sham TEAS group: Electrodes will be placed in the same manner as the perioperative TEAS group but without electrical stimulation. Participants will be informed that the treatment has no sensation.

Two nursing postgraduates, who will serve as interveners, are required to spend 2 months in the Traditional Chinese Medicine Department of the hospital, dedicating 4 h each day to learning the functions and locations of acupoints, as well as the inspection and use of the instruments. To be eligible to participate in this intervention, the interveners must successfully pass a final examination, which will assess both theoretical knowledge and practical skills, administered by the Traditional Chinese Medicine Department.

During the experiment, a specialist from the Chinese Medicine Department will be invited to assess the appropriateness of acupoint selection and treatment intensity on a weekly basis. If any inaccuracies are identified in either the acupoint selection or treatment intensity, the intervener will be required to rejoin the Traditional Chinese Medicine Department for further training until they can accurately locate the acupoints and determine the correct treatment intensity. If a patient is affected by any such inaccuracies, they will be required to withdraw from the study.

### Safety analysis

All data will be collected by data collectors, consisting of two nursing undergraduates. Any treatment-related adverse events (AEs), such as allergies, fainting, vomiting, prolonged skin numbness, redness, swelling, pain, or other injuries occurring within 24 h after each treatment, will be closely monitored and documented. These observations will be made either through direct observation by interveners or through self-reports by the patients (14). During the intervention process, if the occurrence of AEs exceeds 5%, we will hold discussions and make appropriate adjustments to the research protocol. Once identified, all AEs related to treatment will be covered by the research group.

### Outcome measures

#### Main outcome

The primary outcome of this study will be the change in cognition. The neuropsychological test will be measured preoperatively and 3 days postoperatively. Telephone Interviews for Cognitive Status-Modified (TICS-m) will be measured 1, 3, and 6 months after the operation.

The neuropsychological test includes the Mini-Mental State Examination (MMSE), Auditory Verbal Learning Test-HuaShan version (AVLT-H), Digital Symbol Coding (DSC), Verbal Fluency Test (VFT), and Clock Drawing Task (CDT). These assessments will be conducted by experienced researchers who have received training in neuropsychological assessment. Postoperative cognitive dysfunction (POCD) is defined as occurring when two or more Test *Z*-values or the *Z*-total exceed 1.96, according to the “Z-score method” (31).

The MMSE is a widely used tool for detecting cognitive impairment in both clinical and research settings (32). This 30-point questionnaire evaluates various cognitive domains, including orientation (time and place), memory (immediate and short-term), attention, calculation, and language (naming, repetition, listening, reading, and writing) (33). A higher score indicates better cognitive function.

AVLT-H has been shown to be acceptable to Mandarin speakers and is sensitive to detecting amnestic mild cognitive impairment (aMCI) (34). It assesses memory by requiring patients to learn and recall a set of 12 words. Due to the knowledge and cooperation levels of the local elderly subjects, we will record their fourth recall, with each correctly recalled word earning 1 point (35).

DSC assesses executive function. In this test, subjects are required to match numbers with corresponding symbols within 90 s. Each correctly matched number earns 1 point (36). This test is a valid and reliable measure for detecting early signs of cognitive decline (37) and predicting cognitive disorders (38).

VFT primarily assesses language ability but also requires the integration of several cognitive functions, including speed, attention, inhibition, and self-monitoring (39). Although the task is relatively simple, it reflects the effective coordination of these functions (40). In the test, subjects are instructed to name as many animals, fruits, and vegetables as they can within 1 min. Each correctly named item earns 1 point, and repeated items receive no points (41).

CDT is a sensitive and specific measure for cognitive impairment (42) that evaluates visual–spatial structural ability. In this test, subjects are required to draw the dial of a clock on paper and correctly place the numbers in their respective positions. Scoring is as follows: ① Drawing a closed circular outline earns 1 point. ② Displaying the correct numbers in the proper sequence on the dial earns 2 points. ③ Positioning the hour and minute hands correctly earns 1 point. The test times are 08:10 (pre-) and 07:50 (post-) (43).

TICS-m is modeled after the MMSE and comprises 21 items categorized into three dimensions, with a total score of 50 points: memory (20 points), orientation (13 points), and language and attention (17 points) (44). A score of <28 points is indicative of dementia, while a score of 28–32 points is considered mild cognitive impairment (MCI). TICS-m has demonstrated good reliability and validity in the Chinese population (39).

#### Secondary outcome

Secondary outcomes will include measurements of Interleukin-6 (IL-6), S100 calcium-binding protein β (S100β), insulin-like growth factor-1 (IGF-1), tumor necrosis factor (TNF-*α*), and C-reactive protein (CRP) both before and on the third day after the operation. These factors are significantly elevated and closely associated with POCD (45–49). S100β, present in glial and Schwann cells, increases in blood and cerebrospinal fluid with brain damage and serves as a marker for POCD (50). IL-6 is a proinflammatory cytokine involved in CNS inflammation (51). IGF-1 protects dopaminergic neurons and delays their degeneration, particularly in midbrain regions (52). TNF-*α*, a key proinflammatory cytokine, is associated with autoimmune and cognitive disorders (53). CRP, a non-specific inflammatory biomarker, accelerates neurodegenerative diseases by activating microglia and proinflammatory pathways (49). IL-6, IGF-1, TNF-α, and S100β will be quantified using commercial ELISA kits (Beyotime, China), while CRP data will be extracted from the case records.

We will also gather postoperative data, including information on exhaustion, defecation and eating times, and the time of the first postoperative ambulation. Additionally, Numeric Rating Scale (NRS) scores will be collected before and on the third day after the operation, along with Activities of Daily Living (ADL) and Braden scale scores taken before the operation and at the time of discharge from the case records.

### Data collection and management

Throughout the study, participants will be referred to by their participant numbers rather than by their names. Additionally, all relevant documents and files will be archived for a period of 5 years and will only be accessible to investigators who have signed a confidentiality agreement or to institutional or governmental auditors during the study. Data collection and management will be monitored by the Institutional Ethics Committee of Subei People’s Hospital, Jiangsu Province.

### Statistical analysis

All data will be analyzed using IBM SPSS software version 24.0. Research data following a normal distribution and expressed as x̄± SD will be analyzed using analysis of variance (ANOVA). For non-normally distributed data expressed as median (interquartile range), the Kruskal-Wallis H test will be applied. Categorical variables, presented as frequency (f) and percentages (%), will be analyzed using the chi-squared test and Fisher’s exact test as appropriate. Multiple imputation methods will be employed to handle missing data.

## Discussion

Research has shown that the timing of TEAS interventions—whether administered 10–30 min preoperatively, throughout the entire surgical procedure, or solely preoperatively versus a combination of preoperative and postoperative treatment—can significantly improve cognitive function (12–14). Extended use of TEAS may lead to a “tolerance effect,” potentially diminishing its efficacy over time (19). Consequently, this protocol aims to investigate the optimal timing for TEAS to improve postoperative recovery in elderly patients. The results are expected to assist clinicians in formulating more effective perioperative management strategies, reducing the incidence of POCD, and enhancing postoperative cognitive function and quality of life for elderly patients.

Additionally, given the potential for a “tolerance effect” associated with prolonged TEAS use, it is essential to examine how varying intervention durations at different timings might influence this phenomenon. Future research should evaluate the impact of different intervention durations at these timings to ensure the sustained efficacy and safety of TEAS. Furthermore, exploring the effects of TEAS across diverse surgical procedures and patient subgroups will contribute to validating its generalizability and applicability. Further studies are needed to confirm the long-term effects and safety of TEAS to establish its reliability in clinical practice.

This study has several notable strengths. Firstly, it is the pioneering investigation into the effects of different TEAS timings on the postoperative recovery of elderly patients with gastrointestinal tumors, as previously mentioned. Second, we assessed the long-term cognitive function. Many studies have discussed TEAS in the short term, for example, in the third and seventh days after the operation (32, 45). However, POCD can last for months or years, and we investigated the cognitive function of patients 1, 3, and 6 months postoperative (6).

Third, we included inflammatory factors such as S100β, IL-6, IGF-1, and CRP, which have been reported to be upregulated in patients with POCD, in order to investigate the impact of TEAS on POCD in our patient cohort. S100β protein concentrations, primarily found in glial and Schwann cells as the β subunit of a calcium-binding protein, tend to increase in both human blood and cerebrospinal fluid following a variety of diseases or conditions associated with brain damage (50). Numerous studies have demonstrated the predictive value of S100β for POCD (54). IL-6 is a proinflammatory cytokine mediating inflammatory and immune responses in the CNS (51). As one of the neurotrophic factors, IGF-1 has been proven to have neuroprotective and neuroproliferative effects. It typically exerts its protective effects on dopaminergic neurons, delaying their degeneration and providing protection, particularly in midbrain regions like the ventral tegmental area (VTA) and the substantia nigra (52, 55). Tumor necrosis factor-*α* (TNF-α) is a pivotal proinflammatory cytokine that plays a central role in the regulation of both innate and adaptive immunity (1). TNF-α is known to be closely associated with a range of autoimmune and neurological disorders, including cognitive decline (53). CRP is a non-specific inflammatory biomarker that has been proven to accelerate the development of neurodegenerative disorders by activating microglia, increasing levels of proinflammatory cytokines, and activating the complement cascade (49).

Additionally, we investigated the differences in Activities of Daily Living (ADL) and Braden scale scores both before the operation and at the time of discharge by reviewing the case records in different groups. In a previous study, we observed significant differences in Braden scale scores at the time of discharge between the pre- and postoperative TEAS group and the control group, while ADL scores remained similar (18). However, few studies have analyzed the impact of different timings of TEAS on ADL and Braden scale scores before and on the third day after the operation. This study aims to provide valuable insights into the specific effects of TEAS at various time points.

Furthermore, some studies have explored the impact of TEAS on factors such as exhaustion, defecation, eating, and the time taken to get out of bed for the first time, as well as perioperative Numeric Rating Scale (NRS) scores. However, limited research has focused on determining the most suitable timing for TEAS to enhance the postoperative recovery of patients. Therefore, this study is expected to suggest an optimal time window for expediting the recovery of patients with gastrointestinal tumors.

It is important to acknowledge certain limitations in this protocol. Due to the low outpatient follow-up rate observed in previous investigations, we were limited to using the TICS-m scale to assess long-term cognitive function post-operation, which differs from the previous neuropsychological tests. This limitation may introduce some bias, as it only allows for the detection of cognitive impairment without the ability to directly compare long-term cognitive function before and after the operation. Furthermore, as this is a single-center study, the results may not be fully generalizable to other research centers or populations, limiting the external validity of our findings. To address these limitations, future studies should consider multi-center designs to increase the diversity of the patient sample and enhance the applicability of the results across different settings. Additionally, expanding the range of neuropsychological assessments used to evaluate cognitive function, along with incorporating multiple testing points in longitudinal studies, would provide a more comprehensive and nuanced understanding of cognitive changes post-operation. These improvements would contribute to refining the research design and furthering our understanding of cognitive recovery in this context.

## Glossary

TEAS: Transcutaneous acupoint electrical stimulation
POCD: Postoperative cognitive dysfunction
TICS-m: Telephone Interview for Cognitive Status-Modified
IL-6: Interleukin-6
S100β: S100 calcium proteinβ
IGF-1: Insulin-like growth factor-1
CRP: C-reactive protein
NRS: Numeric rating scale
ADL: Activities of Daily Living
ANOVA: Analyses of variance
TENS: Transcutaneous electrical nerve stimulation
MMSE: Mini-Mental State Examination
ASA: American Society of Anesthesiology
AVLT-H: Auditory Verbal Learning Test-HuaShan version
DSC: Digital Symbol Coding
VFT: Verbal Fluency Test
CDT: Clock Drawing Task
aMCI: Amnestic mild cognitive impairment
MCI: Mild cognitive impairment
AEs: Adverse events
CNS: Central nervous system

## References

[1] Zeng Y Xia J Chen Z Tian X Ren Y. Transcutaneous electrical acupoint stimulation (TEAS) for cancer-related fatigue: study protocol for a systematic review and meta-analysis. BMJ Open. (2021) 11:e049318. doi: 10.1136/bmjopen-2021-049318, PMID: 34819280 PMC8614145

[2] Feng B Zhang Y Luo LY Wu JY Yang SJ Zhang N . Transcutaneous electrical acupoint stimulation for post-traumatic stress disorder: Assessor-blinded, randomized controlled study. Psychiatry Clin Neurosci. (2019) 73:179–86. doi: 10.1111/pcn.12810, PMID: 30565342

[3] Kristek G Radoš I Kristek D Kapural L Nešković N Škiljić S . Influence of postoperative analgesia on systemic inflammatory response and postoperative cognitive dysfunction after femoral fractures surgery: a randomized controlled trial. Reg Anesth Pain Med. (2019) 44:59–68. doi: 10.1136/rapm-2018-000023, PMID: 30640654

[4] Deiner S Liu X Lin HM Jacoby R Kim J Baxter MG . Does postoperative cognitive decline result in new disability after surgery? Ann Surg. (2021) 274:e1108–14. doi: 10.1097/SLA.0000000000003764, PMID: 32149824

[5] Olotu C. Postoperative neurocognitive disorders. Curr Opin Anaesthesiol. (2020) 33:101–8. doi: 10.1097/ACO.0000000000000812, PMID: 31764008

[6] Evered LA Silbert BS. Postoperative cognitive dysfunction and noncardiac surgery. Anesth Analg. (2018) 127:496–505. doi: 10.1213/ANE.0000000000003514, PMID: 29889707

[7] Borges J Moreira J Moreira A Santos A Abelha FJ. Impact of postoperative cognitive decline in quality of life: a prospective study. Rev Bras Anestesiol. (2017) 67:362–9. doi: 10.1016/j.bjan.2016.07.007, PMID: 28412051

[8] Liu J Huang K Zhu B Zhou B Ahmad Harb AK Liu L . Neuropsychological tests in post-operative cognitive dysfunction: methods and applications. Front Psychol. (2021) 12:684307. doi: 10.3389/fpsyg.2021.684307, PMID: 34149572 PMC8212929

[9] Xi L-J Fang F . Research progress on the effect of percutaneous acupoint electrical stimulation on postoperative neurocognitive function. Nurs Res. (2021) 35:2163–7. doi: 10.12102/j.issn.1009-6493.2021.12.018

[10] Huang X Zhang J-X Luo T Wei C-w Wu A-S. Effect of percutaneous acupoint electrical stimulation on postoperative neurocognitive function in patients undergoing non-stop coronary artery bypass grafting. J Clin Anesthes. (2020) 36:861–5. doi: 10.12089/jca.2020.09.006

[11] Wu X-H Chen W-T. Effects of percutaneous acupoint electrical stimulation assisted general anesthesia on postoperative immune function and cognitive function in elderly patients undergoing heart surgery. China Med Herald. (2019) 16:151–4.

[12] Guo F Han R Sun L Zheng L Wang Y Yan Y . Effect of transcutaneous electrical acupoint stimulation on postoperative cognitive function in older patients with lung cancer: a randomized, double-blind, placebo-controlled trial. Heliyon. (2023) 9:e19386. doi: 10.1016/j.heliyon.2023.e19386, PMID: 37809441 PMC10558345

[13] Wang LF Liang WD Wang BY Guo ML Zhou JS Chen L . Transcutaneous electrical acupoint stimulation for reducing cognitive dysfunction in lumbar spine surgery: a randomized, controlled trail. Front Aging Neurosci. (2022) 14:1034998. doi: 10.3389/fnagi.2022.1034998, PMID: 36545028 PMC9760873

[14] Xi L Fang F Yuan H Wang D. Transcutaneous electrical acupoint stimulation for postoperative cognitive dysfunction in geriatric patients with gastrointestinal tumor: a randomized controlled trial. Trials. (2021) 22:563. doi: 10.1186/s13063-021-05534-9, PMID: 34425851 PMC8383437

[15] Chang XL Liu XM An LX Zheng JY Zhang K. Effects of transcutaneous electrical acupoint stimulation (TEAS) on postoperative pain in patients undergoing gastric and esophageal ESD surgery: a study protocol for a prospective randomized controlled trial. BMC Complement Med Ther. (2023) 23:253. doi: 10.1186/s12906-023-04075-9, PMID: 37474962 PMC10357617

[16] Lu Z Luo A Min S Dong H Xiong Q Li X . Acupoint stimulation for enhanced recovery after Colon surgery: a prospective multicenter randomized controlled trial. J Multidiscip Healthc. (2022) 15:2871–9. doi: 10.2147/JMDH.S391852, PMID: 36570812 PMC9785190

[17] Li WJ Gao C An LX Ji YW Xue FS Du Y. Perioperative transcutaneous electrical acupoint stimulation for improving postoperative gastrointestinal function: a randomized controlled trial. J Integr Med. (2021) 19:211–8. doi: 10.1016/j.joim.2021.01.005, PMID: 33495134

[18] Tarazona-Santabalbina FJ Gómez-Cabrera MC Pérez-Ros P Martínez-Arnau FM Cabo H Tsaparas K . A multicomponent exercise intervention that reverses frailty and improves cognition, emotion, and social networking in the community-dwelling frail elderly: a randomized clinical trial. J Am Med Dir Assoc. (2016) 17:426–33. doi: 10.1016/j.jamda.2016.01.019, PMID: 26947059

[19] Han J-S. Acupuncture analgesia research. Acupunct Res. (2016) 41:377–87. doi: 10.13702/j.1000-0607.2016.05.001 29071939

[20] Balduino E de Melo BAR de Sousa Mota da Silva L Martinelli JE Cecato JF. The "SuperAgers" construct in clinical practice: neuropsychological assessment of illiterate and educated elderly. Int Psychogeriatr. (2020) 32:191–8. doi: 10.1017/S1041610219001364, PMID: 31556369

[21] Cesari M Penninx BW Pahor M Lauretani F Corsi AM Rhys Williams G . Inflammatory markers and physical performance in older persons: the InCHIANTI study. J Gerontol A Biol Sci Med Sci. (2004) 59:242–8. doi: 10.1093/gerona/59.3.M242, PMID: 15031308

[22] Lu GW Zhang YL Wang TH Gu YQ Lei JQ Cui MM . Assessment of efficacy of acupuncture combined with hyperbaric oxygen therapy for patients with delayed encephalopathy of CO intoxication by magnetic resonance voxel incoherent motion imaging. Zhen Ci Yan Jiu. (2020) 45:407–11. doi: 10.13702/j.1000-0607.180678, PMID: 32447857

[23] Wei Q-L Pang Y-H Zuo H-J Mo X-W W R-Y L X. Effect of percutaneous acupoint electrical stimulation combined with chewing gum on gastrointestinal function in postoperative patients with colorectal cancer. Chin J Modern Nurs. (2019) 25:2746–9. doi: 10.3760/cma.j.issn.1674-2907.2019.21.024

[24] Wang C Liang X Yu Y Li Y Wen X Liu M. Electroacupuncture pretreatment alleviates myocardial injury through regulating mitochondrial function. Eur J Med Res. (2020) 25:29. doi: 10.1186/s40001-020-00431-4, PMID: 32738910 PMC7395969

[25] Tang YW Cui X Liu K Li XX Han S Zhao J . Manual acupuncture stimulation of acupoints at the same and adjacent spinal segments promotes uterine contractility. Zhen Ci Yan Jiu. (2020) 45:708–13. doi: 10.13702/j.1000-0607.200476, PMID: 32959552

[26] Yang H Yang H Wang L Shi H Liu B Lin X . Transcutaneous Neuromodulation improved inflammation and sympathovagal ratio in patients with primary biliary ssscholangitis and inadequate response to Ursodeoxycholic acid: a pilot study. BMC Complement Med Ther. (2020) 20:242. doi: 10.1186/s12906-020-03036-w, PMID: 32738911 PMC7395375

[27] Duan Z-X Wu X-H Wang J-H L F. Effect of percutaneous acupoint electrical stimulation assisted anesthesia on pain and rapid rehabilitation in elderly patients undergoing thoracoscopic surgery. Chin J Geriatr. (2020) 39:323–7. doi: 10.3760/cma.j.issn.0254-9026.2020.03.017

[28] Duan C-Z Xun S-N Zhang X-Q Jiao M-N Guo X-R. Effects of transcutaneous acupoint electrical stimulation pretreatment on postoperative cognitive dysfunction and inflammatory factors in elderly patients. J Ningxia Med Univ. (2021) 43:5. doi: 10.16050/j.cnki.issn1674-6309.2021.01.008

[29] Liu T Yin C Li Y Gao F Yu L Wang Z . Effects of transcutaneous electrical Acupoint stimulation on postoperative cognitive decline in elderly patients: a pilot study. Clin Interv Aging. (2021) 16:757–65. doi: 10.2147/CIA.S309082, PMID: 33976542 PMC8106456

[30] Zhao J-Y Shen H-F. Effect of percutaneous acupoint electrical stimulation on elderly patients with cognitive impairment after anesthesia serum NSE, S100β influence of content. Zhejiang J Tradit Chin Med. (2019) 54:65. doi: 10.13633/j.cnki.zjtcm.2019.08.040

[31] Raffin J Rolland Y Aggarwal G Nguyen AD Morley JE Li Y . Associations between physical activity, blood-based biomarkers of neurodegeneration, and cognition in healthy older adults: the MAPT study. J Gerontol A Biol Sci Med Sci. (2021) 76:1382–90. doi: 10.1093/gerona/glab094, PMID: 33864068

[32] Lim MYL Loo JHY. Screening an elderly hearing impaired population for mild cognitive impairment using Mini-mental state examination (MMSE) and Montreal cognitive assessment (MoCA). Int J Geriatr Psychiatry. (2018) 33:972–9. doi: 10.1002/gps.4880, PMID: 29575215

[33] Jia X Wang Z Huang F Su C du W Jiang H . A comparison of the Mini-mental state examination (MMSE) with the Montreal cognitive assessment (MoCA) for mild cognitive impairment screening in Chinese middle-aged and older population: a cross-sectional study. BMC Psychiatry. (2021) 21:485. doi: 10.1186/s12888-021-03495-6, PMID: 34607584 PMC8489046

[34] Zhao Q Lv Y Zhou Y Hong Z Guo Q. Short-term delayed recall of auditory verbal learning test is equivalent to long-term delayed recall for identifying amnestic mild cognitive impairment. PLoS One. (2012) 7:e51157. doi: 10.1371/journal.pone.0051157, PMID: 23236445 PMC3517417

[35] Lin G Lan F Wu D Cao G Li Z Qi Z . Resting-state functional connectivity alteration in elderly patients with knee osteoarthritis and declined cognition: An observational study. Front Aging Neurosci. (2022) 14:1002642. doi: 10.3389/fnagi.2022.1002642, PMID: 36337709 PMC9634173

[36] Wessels AM Lines C Stern RA Kost J Voss T Mozley LH . Cognitive outcomes in trials of two BACE inhibitors in Alzheimer's disease. Alzheimers Dement. (2020) 16:1483–92. doi: 10.1002/alz.12164 33049114

[37] Donohue MC Sperling RA Salmon DP Rentz DM Raman R Thomas RG . The preclinical Alzheimer cognitive composite: measuring amyloid-related decline. JAMA Neurol. (2014) 71:961–70. doi: 10.1001/jamaneurol.2014.803, PMID: 24886908 PMC4439182

[38] Best JR Liu-Ambrose T Boudreau RM Ayonayon HN Satterfield S Simonsick EM . An evaluation of the longitudinal, bidirectional associations between gait speed and cognition in older women and men. J Gerontol A Biol Sci Med Sci. (2016) 71:1616–23. doi: 10.1093/gerona/glw066, PMID: 27069098 PMC5106856

[39] Sutin AR Luchetti M Stephan Y Strickhouser JE Terracciano A. The association between purpose/meaning in life and verbal fluency and episodic memory: a meta-analysis of >140,000 participants from up to 32 countries. Int Psychogeriatr. (2022) 34:263–73. doi: 10.1017/S1041610220004214, PMID: 33612145 PMC8380267

[40] Shao Z Janse E Visser K Meyer AS. What do verbal fluency tasks measure? Predictors of verbal fluency performance in older adults. Front Psychol. (2014) 5:772. doi: 10.3389/fpsyg.2014.00772, PMID: 25101034 PMC4106453

[41] Li WX Yuan J Han F Zhou LX Ni J Yao M . White matter and gray matter changes related to cognition in community populations. Front Aging Neurosci. (2023) 15:1065245. doi: 10.3389/fnagi.2023.1065245, PMID: 36967830 PMC10036909

[42] Yap PL Ng TP Niti M Yeo D Henderson L. Diagnostic performance of clock drawing test by CLOX in an Asian Chinese population. Dement Geriatr Cogn Disord. (2007) 24:193–200. doi: 10.1159/000107080, PMID: 17690551

[43] Leissing-Desprez C Thomas E Segaux L Broussier A Oubaya N Marie-Nelly N . Understated cognitive impairment assessed with the clock-drawing test in community-dwelling individuals aged ≥50 years. J Am Med Dir Assoc. (2020) 21:1658–64. doi: 10.1016/j.jamda.2020.03.016, PMID: 32387111

[44] Ran J Bai X Wang R Li X. Role of Dexmedetomidine in early POCD in patients undergoing thoracic surgery. Biomed Res Int. (2021) 2021:8652028. doi: 10.1155/2021/8652028, PMID: 34859103 PMC8632391

[45] He X Wen L-J Cui C Li D-R Teng J-F. The significance of S100β protein on postoperative cognitive dysfunction in patients who underwent single valve replacement surgery under general anesthesia. Eur Rev Med Pharmacol Sci. (2017) 21:2192–8. 28537663

[46] Peng L Xu L Ouyang W. Role of peripheral inflammatory markers in postoperative cognitive dysfunction (POCD): a Meta-analysis. PLoS One. (2013) 8:e79624. doi: 10.1371/journal.pone.0079624, PMID: 24236147 PMC3827367

[47] Westwood AJ Beiser A DeCarli C Harris TB Chen TC He XM . Insulin-like growth factor-1 and risk of Alzheimer dementia and brain atrophy. Neurology. (2014) 82:1613–9. doi: 10.1212/WNL.0000000000000382, PMID: 24706014 PMC4013812

[48] Cao Z Min J Tan Q Si K Yang H Xu C. Circulating insulin-like growth factor-1 and brain health: evidence from 369,711 participants in the UK biobank. Alzheimers Res Ther. (2023) 15:140. doi: 10.1186/s13195-023-01288-5, PMID: 37608387 PMC10463341

[49] Vintimilla R Hall J Johnson L O'Bryant S. The relationship of CRP and cognition in cognitively normal older Mexican Americans: a cross-sectional study of the HABLE cohort. Medicine. (2019) 98:e15605. doi: 10.1097/MD.0000000000015605, PMID: 31083252 PMC6531144

[50] Yardan T Cevik Y Donderici O Kavalci C Yilmaz FM Yilmaz G . Elevated serum S100B protein and neuron-specific enolase levels in carbon monoxide poisoning. Am J Emerg Med. (2009) 27:838–42. doi: 10.1016/j.ajem.2008.04.016, PMID: 19683113

[51] Kim YS Lee KJ Kim H. Serum tumour necrosis factor-α and interleukin-6 levels in Alzheimer's disease and mild cognitive impairment. Psychogeriatrics: the official journal of the Japanese psychogeriatric. Society. (2017) 17:224–30. doi: 10.1111/psyg.12218, PMID: 28130814

[52] Cohen E. Aging, protein aggregation, chaperones, and neurodegenerative disorders: mechanisms of coupling and therapeutic opportunities. Rambam Maimonides Med J. (2012) 3:e0021. doi: 10.5041/RMMJ.10088, PMID: 23908845 PMC3678828

[53] Ortí-Casañ N Zuhorn IS Naudé PJW de P van P Wajant H . A TNF receptor 2 agonist ameliorates neuropathology and improves cognition in an Alzheimer's disease mouse model. Proc Natl Acad Sci USA. (2022) 119:e2201137119. doi: 10.1073/pnas.2201137119, PMID: 36037389 PMC9482428

[54] Wang X Chen X Wu F Liu Y Yang Y Chen W . Relationship between postoperative biomarkers of neuronal injury and postoperative cognitive dysfunction: a meta-analysis. PLoS One. (2023) 18:e0284728. doi: 10.1371/journal.pone.0284728, PMID: 37098084 PMC10128950

[55] Tong M Dong M de la Monte SM. Brain insulin-like growth factor and neurotrophin resistance in Parkinson's disease and dementia with Lewy bodies: potential role of manganese neurotoxicity. J Alzheimers Dis. (2009) 16:585–99. doi: 10.3233/JAD-2009-0995, PMID: 19276553 PMC2852260

